# Mycobacterial Phylogenomics: An Enhanced Method for Gene Turnover Analysis Reveals Uneven Levels of Gene Gain and Loss among Species and Gene Families

**DOI:** 10.1093/gbe/evu117

**Published:** 2014-06-05

**Authors:** Pablo Librado, Filipe G. Vieira, Alejandro Sánchez-Gracia, Sergios-Orestis Kolokotronis, Julio Rozas

**Affiliations:** ^1^Departament de Genètica and Institut de Recerca de la Biodiversitat (IRBio), Universitat de Barcelona, Barcelona, Spain; ^2^Department of Integrative Biology, University of California, Berkeley; ^3^Department of Biological Sciences, Fordham University; ^4^Sackler Institute for Comparative Genomics, American Museum of Natural History, New York, New York

**Keywords:** gene turnover rates, gene gain and loss, gene families, maximum likelihood, rate heterogeneity, *M. tuberculosis*

## Abstract

Species of the genus *Mycobacterium* differ in several features, from geographic ranges, and degree of pathogenicity, to ecological and host preferences. The recent availability of several fully sequenced genomes for a number of these species enabled the comparative study of the genetic determinants of this wide lifestyle diversity. Here, we applied two complementary phylogenetic-based approaches using information from 19 *Mycobacterium* genomes to obtain a more comprehensive view of the evolution of this genus. First, we inferred the phylogenetic relationships using two new approaches, one based on a *Mycobacterium*-specific amino acid substitution matrix and the other on a gene content dissimilarity matrix. Then, we utilized our recently developed gain-and-death stochastic models to study gene turnover dynamics in this genus in a maximum-likelihood framework. We uncovered a scenario that differs markedly from traditional 16S rRNA data and improves upon recent phylogenomic approaches. We also found that the rates of gene gain and death are high and unevenly distributed both across species and across gene families, further supporting the utility of the new models of rate heterogeneity applied in a phylogenetic context. Finally, the functional annotation of the most expanded or contracted gene families revealed that the transposable elements and the fatty acid metabolism-related gene families are the most important drivers of gene content evolution in *Mycobacterium*.

## Introduction

The genus *Mycobacterium* represents a large group of approximately 120–170 ecologically diverse type strains (American Type Culture Collection, http://www.atcc.org, last accessed June 12, 2014; Leibniz Institute DSMZ-German Collection of Microorganisms and Cell Cultures, http://www.dsmz.de; StrainInfo, http://www.straininfo.net/taxa/1059, last accessed June 12, 2014) that include both animal-adapted and free-living taxa. Although most of these species are ubiquitous environmental saprophytes, around one-third are pathogenic to vertebrates (Howard and Byrd 2000). These include members of the *Mycobacterium tuberculosis* complex (MTBC), such as *M. tuberculosis* (the etiologic agent of tuberculosis and the major cause of death in AIDS patients worldwide) (Raviglione et al. 1995), *M. bovis* (causes tuberculosis in cattle and other mammals) (O’Reilly and Daborn 1995; Une and Mori 2007) or *M. africanum* (infects humans specifically in West Africa) (de Jong et al. 2010), as well as *M. leprae* (the causative agent of leprosy) (Sasaki et al. 2001), *M. ulcerans* (responsible for the Buruli ulcer) (van der Werf et al. 1999) or the *M. avium* complex, consisting of *M. avium* (opportunistic human pathogen) and *M. avium* ssp. *paratuberculosis* (causes Johne’s disease in ruminants; see Johne’s Information Center, University of Wisconsin, at http://www.johnesdisease.org and http://www.johnes.org, last accessed June 12, 2014), among others.

In recent years, there has been an emergence of tools and techniques for the rapid characterization of complete microbial genomes (MacLean et al. 2009; Comas et al. 2010). At present, we are able to exploit the powerful analytical methods of molecular evolution and population genomics to determine the relative contribution of the different evolutionary forces that shape mycobacterial genome organization, structure, and diversity. These methods offer the exceptional opportunity to explore the genetic and genomic determinants of pathogenesis, virulence, or lifestyle diversity in bacteria. Moreover, such analyses not only contribute to a better understanding of the biology of these species but also improve the diagnosis of mycobacterial diseases and the development of new strategies to identify potential drug targets and vaccine candidates.

The mutational events underlying gene and genome evolution are varied, ranging from nucleotide substitutions, to gene gains and losses, or changes in genomic structure and organization. Comparative genomic studies in the *Mycobacterium* genus have uncovered a genomic evolution characterized by frequent and unevenly distributed gene gain-and-loss events, being some of them associated with pathogenesis and virulence. Nevertheless, these studies either applied simple pairwise comparisons of genomic features (Gomez-Valero et al. 2007) or used the gene tree and species tree reconciliation approach under a parsimony context (McGuire et al. 2012). Therefore, the use of full probabilistic approaches that explicitly take heterogeneity into account (both across gene families and lineages) can provide robust parameter estimates (gene turnover rates, ancestral gene content, number of gene gains and loss events, etc.) and a rigorous statistical framework to contrast the fit of different biologically realistic scenarios. Nevertheless, the performance of maximum-likelihood (ML) approaches in a phylogenetic context is highly dependent on the accuracy of the underlying species tree. When the tree is not simultaneously coestimated, the analysis should be performed applying the most accurate topology and branch lengths. In recent years a number of phylogenomic analyses have studied the phylogenetic relationships on the genus *Mycobacterium* (Vishnoi et al. 2010; McGuire et al. 2012; Smith et al. 2012; Prasanna and Mehra 2013). However, these results do not agree on a single evolutionary history for the diversification of the genus, showing differences in both topology and branch lengths.

Here, we have estimated the phylogenetic relationships of the genus using two new approaches: One based on a *Mycobacterium*-specific amino acid substitution matrix inferred from the protein-based alignments of 19 genomes and the other based on gene content dissimilarity. Taking advantage of this, we performed an exhaustive study of the gain-and-death (GD) dynamics, with special focus on gene turnover rate heterogeneity both across lineages and among families. The results uncover an evolutionary scenario that differs, to some extent, from most previously published molecular systematics studies although it is in agreement with those who advocate that the position of *M. leprae* as a sister taxon to the MTBC. We also show that mycobacterial evolution has been dominated by a high gene gain-and-loss dynamic with strong heterogeneity across lineages and gene families, especially for those families involved in transposition and fatty acid biosynthesis.

## Materials and Methods

### Mycobacterial Genomes and Orthologous Gene Identification

We retrieved the genome sequences of several *Mycobacterium* species publicly available on the Integr8 database (Kersey et al. 2005) (supplementary table S1, Supplementary Material online), including their protein sequences and corresponding pairwise orthologous relationships. We included a total of 19 strains in this study: *M. tuberculosis* H37Rv, *M. tuberculosis* H37Ra, *M. tuberculosis* CDC1551, *M. bovis* BCG/Tokyo 172, *M. bovis* BCG/Pasteur 1173P2, *M. bovis* AF2122197, *M. leprae* TN, *M. leprae* Br4923, *M*. *marinum* M, *M. ulcerans* Agy99, *M. avium* 104, *M. paratuberculosis* K-10, *M.* sp. JLS, *M.* sp. KMS, *M.* sp. MCS, *M. vanbaaleni* PYR-1, *M. gilvum* PYR-GCK, *M. smegmatis* MC2 155, and *M. abscessus* ATCC 19977. To identify groups of orthologous sequences (herein referred as gene families), we clustered the pairwise orthologous relationships using a Markov Clustering Algorithm (Enright et al. 2002) with an inflation value = 1.50.

### Estimation of Substitution Matrices

The multiple sequence alignment for each group of 1:1 orthologous protein sequences (one single gene copy per species; data set Myc19; supplementary table S1, Supplementary Material online) was built with MAFFT 6.603b using the L-INSi algorithm (–localpair –maxiterate 1000) (Katoh et al. 2005). The multiple sequence alignment of the corresponding coding sequence was performed according to a previous study (Vieira et al. 2007) by back-translating the amino acid alignment using in-house developed Perl scripts.

The general time-reversible (GTR or REV) amino acid substitution matrix (Lanave et al. 1984) of the protein alignments of 1:1 orthologs from the 19 genomes was independently estimated using an ML approach so that the time-reversibility condition *f_i_q_ij_* = *f_j_q_ji_* was satisfied, where *f* is the amino acid equilibrium frequency and *q* is the exchangeability rate. A tree topology was estimated in the fine-grained parallel POSIX-threads build of RAxML versions 7 and 8 (Stamatakis 2006; Stamatakis and Ott 2008). First, we used a stepwise-addition maximum parsimony starting tree; subsequently, an ML subtree-pruning-regrafting tree search was performed with the WAG amino acid substitution matrix (Whelan and Goldman 2001) (ten searches). This tree and the WAG matrix were used as a fixed topology and as initial set of substitution rates, respectively, for GTR matrix optimization in the “codeml” program of the PAML 4.3 package (Yang 2007).

### Phylogenetic Analysis

The phylogenetic relationships among mycobacterial genomes were estimated using the ML approach in RAxML, the newly generated GTR matrix (hereafter referred to as MYC for this specific amino acid data set) and by accounting for among-site substitution rate heterogeneity by means of a discrete gamma distribution with four rate categories (Γ_4_) (Yang 1994). Node support was evaluated with 500 bootstrap pseudoreplicates (Felsenstein 1985). To assess the fit of static replacement matrices commonly used for bacterial genomes, we performed the phylogenetic reconstruction in RAxML using the WAG, JTT (Jones et al. 1992), and LG (Le and Gascuel 2008), including the Γ_4_ model with observed amino acid frequencies (+F). The impact of substitution model choice in phylogenetic inference was quantified by comparing their log-likelihoods. A partitioned phylogenetic analysis was also carried out in RAxML by using the same substitution matrix across partitions while unlinking the amino acid frequencies, rate heterogeneity (different Γ distribution *α* shape parameter), and branch lengths (constrained to be proportional among partitions). We also inferred the ML phylogenetic tree of the 19 strains from 16S rRNA sequences in RAxML, mined from the genome assemblies of the examined taxa, and aligned with MAFFT using the L-INSi algorithm.

In order to explore the putative topological discordance among trees of individual loci, we built ML trees for every set of 1:1 protein orthologs. We subsequently constructed a consensus network (Holland et al. 2004) in SplitsTree 4.13.1 (Huson and Bryant 2006) with edge weights reflecting the number of trees containing that edge and a threshold of 10% (i.e., the splits used to build that consensus network existed in at least 10% of the individual trees). We used our estimated MYC amino acid substitution matrix along with empirical amino acid frequencies calculated from every protein alignment. Individual orthologous protein tree support (PTS) was quantified by filtering the phylogenomic ML tree through the swarm of 1,011 individual protein trees. This tree filtering procedure uses a reference tree (the estimated ML tree in this case) as a topological constraint against which other trees (e.g., bootstrap trees or gene trees) can be matched. The proportions of splits/bipartitions that match those of the reference tree are reported as internode branch support values. Tree-to-tree distances were estimated using the Robinson–Foulds (RF) metric (Robinson and Foulds 1981), as implemented in RAxML. Random unrooted trees (*n* = 5,000) for 19 taxa were simulated in T-REX (Boc et al. 2012).

### Gene Content Dissimilarity

We used gene content dissimilarity to generate a distance-based phylogenetic tree. The proportion of mutually exclusive genes between taxa *a* and *b* (genes present in only one taxon in pairwise comparisons) is given by

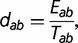

where *E_ab_* is the total number of genes exclusive to taxon *a* and *b*, and *T_ab_* is the total number of genes of these taxa. Operationally, we compute *E*_ab_ as 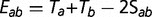 and *T_ab_* as 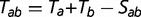, where *T_a_*, *T_b_,* and *S_ab_* are the total number of genes in taxon *a*, in taxon *b*, and the number of shared genes between these taxa, respectively. We clustered taxa based on their *d_ab_* pairwise distances using an improved neighbor-joining algorithm, BioNJ (Gascuel 1997). Bootstrap analysis was performed by randomly sampling (with replacement) the gene families from the original data set. Node support values were calculated using SumTrees (Sukumaran and Holder 2010) from 500 bootstrap replicates.

### Gene Family Evolution

**Fig. 1.—:**
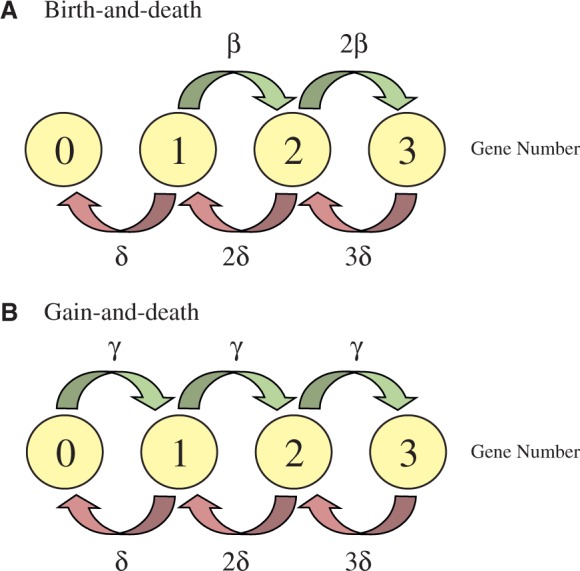
Representation of first four states of two mathematical models of gene family evolution. (*A*) Classical birth-and-death model, where the probability of having a gene gain (birth rate = β) or loss (death rate = δ) event is proportional to the actual number of genes (i.e., density-dependent mechanism). This model is appropriate to study eukaryote gene families, where gene acquisitions mainly result from unequal crossing over, and gene losses from deletion or pseudogenization events. (*B*) GD model, where the probability of having a gain (gain rate = γ) is independent on the actual number of genes (i.e., density-independent mechanisms). This model can be more appropriate for families where genes may originate from molecular processes other than unequal crossing over (e.g., HGT events).

In eukaryotes, the evolution of moderate size families is often modeled by the so-called birth and death (BD) process (Nei and Rooney 2005; Han et al. 2013). This model, appropriate to account single-gene duplications, might not be suitable to analyze gene turnover rates in organisms that may undergo horizontal gene transfer (HGT) events. To circumvent this limitation, we applied the GD stochastic model, as implemented in the BadiRate software (Librado et al. 2012). Unlike the birth process in the BD model, gains in the GD model (fig. 1) can accommodate all kind of gene acquisitions, regardless of their origin (including those by HGT).

**Fig. 2.—:**
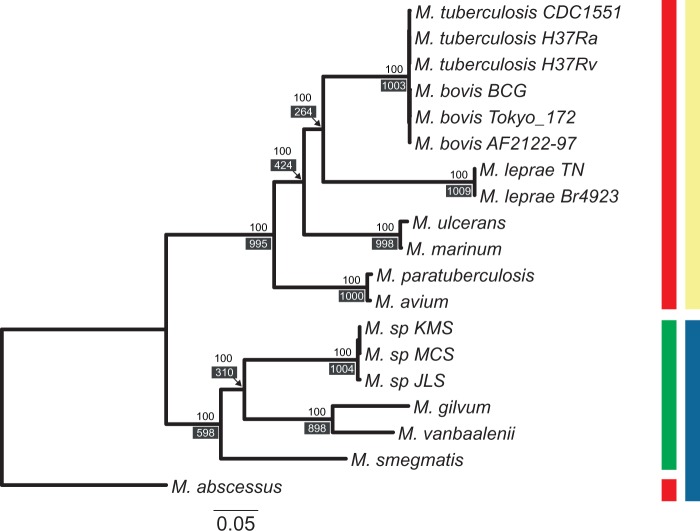
ML phylogenetic reconstruction of the relationships among the 19 *Mycobacterium* taxa inferred from one thousand and eleven 1:1 orthologous protein sequences. Phylogenomic tree based on the MYC substitution matrix estimated from the concatenation of 1,011 mycobacterial orthologs. Each ortholog partition was allowed to evolve under a different Γ_4_ model of among-site rate heterogeneity and its individual observed amino acid frequencies. Red and green bars indicate pathogenic and nonpathogenic mycobacteria, respectively, whereas yellow and blue bars denote slow and rapid growth species, respectively. Values on the nodes indicate the bootstrap support values expressed as the proportion (%) of bootstrap trees that agree with a given bipartition on the best ML tree. Values in dark boxes indicate the number of orthologous protein trees (i.e., individual trees) that agree with a given bipartition on the concatenated alignment-based ML tree. *Mycobacterium abscessus*, which can cause sporadic lung disease, was used as the outgroup.

The analysis was conditioned on the rooted ultrametric tree (by using the *M. abscessus* lineage as ancestor of all other *Mycobacterium* species), obtained from our estimated phylogenomic ML tree (fig. 2) by using nonparametric rate smoothing (Sanderson 1997). We fitted four different branch models to our data (Myc18 data set; supplementary table S1, Supplementary Material online): 1) A global rates (GD-GR-ML) model, where both the gain and death turnover rates are constant over time; 2) a free-rates (GD-FR-ML) model that assumes separate GD rates for each branch; 3) a pathogen-specific rates model (GD-PR-ML), which allows for two branch classes (one for pathogenic and another for nonpathogenic lineages); and, finally, 4) the PR model (GD-PRl-ML) in which the branch leading to the *M. leprae* clade has its own GD rates. In addition, we also fitted a free rates (FR) model that takes into account GD heterogeneity across gene families using two discrete Γ distributions with two categories (GD-FR-ML+dG2). The goodness of fit of these models was assessed using likelihood-ratio tests (LRT) and the Akaike Information Criterion (AIC) (Akaike 1974). We used the best-fit branch model to estimate the gene family gain (γ) and death (δ) rates, as well as the joint reconstruction of the most likely ancestral gene content. We examined the function of the families significantly departing from the overall turnover rates, including a gene ontology (GO) term enrichment analysis as implemented in Ontologizer 2.0 (Bauer et al. 2008).

## Results

### Amino Acid-Based Phylogenetic Tree

The ML phylogenetic tree of the 19 mycobacteria was estimated using information of one thousand and eleven 1:1 orthologs (364,491 amino acids; fig. 2) and our estimated MYC-GTR amino acid substitution matrix (supplementary fig. S1, Supplementary Material online). Overall, it is in concordance with the major relationships known for this genus (Tortoli 2003): MTBC is sister to *M. leprae* with close affinities to *M. marinum* and *M. ulcerans*, and the two *M. avium* strains follow in a ladderized tree order. A sister clade to all the above comprises the rapid-growing, nonpathogenic mycobacteria *M. vanbaalenii*, *M. gilvum* and *M. smegmatis*, as well as the free-living environmental strains.

These relationships are markedly different from those obtained on the 16S rRNA gene phylogeny (supplementary fig. S2, Supplementary Material online). Although 16S rRNA supports the monophyly of slow and rapid growers at 82% and 100%, it provides weak to moderate bootstrap support for middepth nodes, such as the monophyletic *M. leprae*+*M. avium* clade, and the MTBC+(*M. leprae*+*M. avium*) relationship (<66%). Similarly, low levels of support are evident for the placement of *M. smegmatis*, *M. vanbaaleni*, and *M. gilvum* (52–65%). Copy number variation for this gene has been reported at both the intraspecific (Acinas et al. 2004) and interspecific (Vetrovsky and Baldrian 2013) levels. Here, we found slow growers to harbor a single 16S rRNA gene copy, whereas rapid growers contained two copies that were physically separated by well over 1 Mb of their assembled genomes. Copies were mostly identical within species with the exception of *M. vanbaalenii* and the free-living soil strains JLS, KMS, and MCS (supplementary fig. S2, Supplementary Material online). However, using one versus both 16S rRNA gene copies did not impact phylogenetic inference or node stability, with the sole exception of the free-living strains that formed paraphyletic assemblages within their clade, due to extreme sequence conservation in that lineage.

Because the amino acid substitution matrix can also impact the phylogenetic inference (Le and Gascuel 2008), we estimated the specific amino acid replacement matrix for the 19 *Mycobacterium* proteins data set (MYC matrix; supplementary fig. S1, Supplementary Material online). The specific MYC matrix has important differences with respect to the WAG matrix, both in amino acid equilibrium frequencies and in replacement rates. Indeed, the MYC matrix has a higher frequency of Ala, Arg, Cys, and Val and a lower frequency of Asn, Ile, Lys, Phe, Ser, and Tyr. Most exchangeability rates are highly correlated between MYC and WAG, and most differences were owed to radical changes that were more frequent in MYC. Yet, the trees that were built using MYC, WAG, LG, and JTT exhibit identical topologies although the MYC-based tree has a better fit to the data (likelihood scores: −2,947,171 for MYC vs. −2,960,115 for LG+F, −2,966,562 for JTT+F, −2,962,299 for WAG+F). Partitioning the alignment into 1,011 loci yielded an even better likelihood score of −2,937,575.

**Fig. 3.—:**
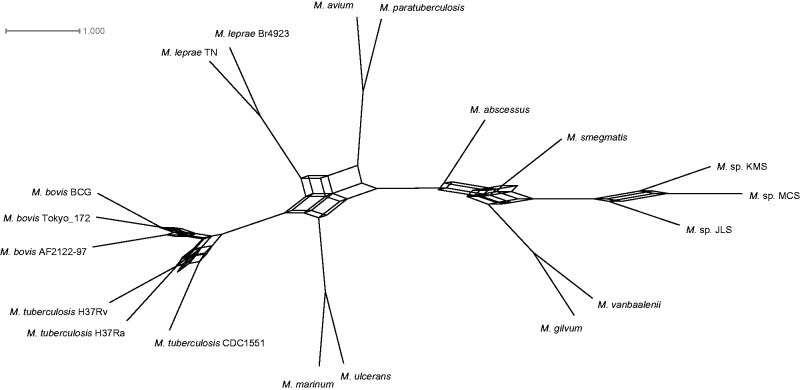
Consensus network built with the 1,011 individual ML amino acid-based trees with a 10% threshold. Scale bar expresses the number of trees containing a given split.

Internal edges on the consensus network of 1,011 protein ML trees showed reticulation, thus indicating disagreement among splits (fig. 3). Although the overall phylogenetic structure of the network is similar to that of the phylogenomic ML, there seems to be localized incongruence (fig. 2). Specifically, for up to a 25% threshold of splits reticulation this incongruence is still apparent (results not shown), thus providing uncertainty on the bifurcating relationships among the MTBC and *M. leprae* (PTS = 264), whereas 366 protein trees support an *M. marinum*+*M. ulcerans*–MTBC relationship, and 253 trees favor an *M. leprae–M. avium* relationship. Similarly, a reticulation remains among the free-living strain group, *M. gilvum*+*M. vanbaalenii*, and *M. smegmatis* with the two alternative groupings being equally supported by orthologs (PTS = 310 for MCS-KMS-JLS–*M. gilvum*+*M. vanbaalenii*, and PTS = 296 for *M. smegmatis–*MCS-KMS-JLS). Once our threshold became more stringent, that is, 30%, the reticulations disappeared and a bifurcating topology emerged (results not shown). Interestingly, this topology differs from the phylogenomic one, as 366 trees favor an MTBC–*M. marinum*+*M. ulcerans* grouping. This indicates that the individual trees causing topological conflict express the history of a minority. Overall, bipartition conflict was variable with only 12 of 510,555 total pairwise protein tree comparisons exhibiting no disagreement and, on the other end of the conflict spectrum, 997 comparisons with trees differing at every split.

These tree support levels were also noticeable when we contrasted the phylogenomic tree to all gene trees (fig. 2). However, this method of tree agreement quantification does not report the alternative splits that are in conflict with the reference—in this case the phylogenomic topology. In order to harvest information on the alternative splits, for example, second and third most prevalent, consensus network approaches are needed. In spite of the topological conflict among protein trees and between the phylogenomic tree and protein trees, the orthologs examined here appear to have related genealogical histories overall, as indicated by the low average relative RF distance of 0.46 (i.e., any two protein trees differed by 14.80 ± 3.25 of 32 splits on average) compared with the RF distance of 0.989 calculated from 5,000 simulated random 19-taxon trees.

### Gene Content Dissimilarity-Based Tree

**Fig. 4.—:**
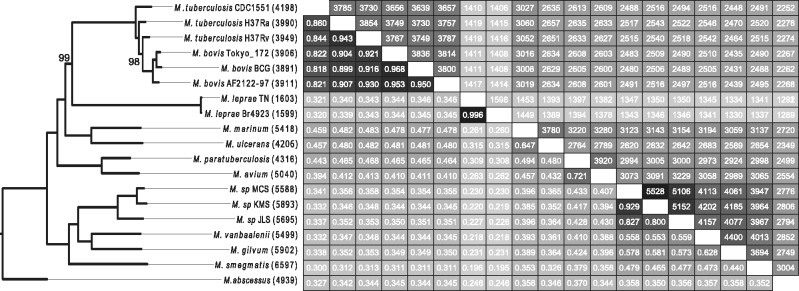
Gene content differences among 19 mycobacterial genomes. The upper diagonal matrix contains the number of genes shared between pairs of genomes, whereas the lower diagonal shows gene content similarities. The neighbor-joining phylogram on the left clusters mycobacterial taxa by gene content dissimilarity. All nodes received 100% bootstrap support except for those denoted on the tree.

The evolutionary dynamics of gene gain and loss and amino acid replacements in orthologous genes can reflect different aspects of genome evolution, especially in the case of mycobacteria. The number of identifiable 1:1 orthologs when considering the 19 genomes is very low (an effect enlarged by the inclusion of a genome with a radically different number of genes, as *M. leprae*), revealing the importance of gene turnover in these species. Then, the information based on the amino acid replacements among orthologs may provide only a partial view of the evolution of the genus. To explore the effect of gene content on the phylogenetic relationships among mycobacteria, we devised a method to capture gene content dissimilarity as a distance measure (see Materials and Methods). Interestingly, gene content-based analysis (fig. 4), which makes use of a different type of genetic information, recapitulates the same topology that the ML phylogenomic protein sequence analysis based on our newly estimated MYC amino acid replacement matrix, except for the MTBC strains. In fact, the analysis showed an increased resolution between close relationships, especially among the strains of this complex. In particular, the H37Rv genome exhibited the highest gene content dissimilarity with respect to the other members of the clade, and the analysis uncovered a ladder-like array of relationships that correlates with the total number of genes as well as corroborates the emergence of *M. bovis* out of *M. tuberculosis* (with a 98% of bootstrap support for this node) (Smith et al. 2006). *Mycobacterium bovis* strains are in fact more closely related to H37Rv than to other *M. tuberculosis* strains (100% of bootstrap support). As expected the gene content-based tree clearly reflects the massive gene loss in the two *M. leprae* strains, which are a very special case for its endosymbiotic life-style (with only 1,603 and 1,599 genes). Moreover, the comparison of gene number between *M. tuberculosis* (3,949) and *M. leprae* (1,605) genes along with their respective genome sizes (4.40 and 3.27 Mb, respectively) indicates a marked discrepancy between genome and gene content dynamics (χ^2^ test; *P* = 2.20 × 10^−^^16^), suggesting that gene loss in *M. leprae* was likely driven by pseudogenization events affecting specific gene regions, rather than a general reduction in the genome size.

### Gene Gain-and-Loss Dynamics in the Evolution of Mycobacteria

To gain deeper insights into the major evolutionary mechanisms that act on mycobacterial evolution, we analyzed the distribution of gene gains and losses within a phylogenetic context using the statistical framework provided by BadiRate (Librado et al. 2012). We fixed the tree topology to the tree inferred under our newly estimated MYC matrix and estimated the GD rates, as well as the number of genes in each internal phylogenetic node. Strikingly, the GD turnover rates were not only extremely high but also unevenly distributed among lineages (after excluding unreliable estimates of short branches, the gain rates ranged from 0 to 1.07 gene gains/amino acid substitution, with death rates from 6.00 × 10^−^^3^ to 4.01 losses/gene/substitution; the highest death rate corresponding to *M. leprae*). In fact, the statistical comparison among the four branch-based assessments showed that GD-FR-ML is the model that best fits the observed gene content data (table 1 and supplementary fig. S3, Supplementary Material online). The mere separation between pathogenic and nonpathogenic evolutionary dynamics (GD-PR-ML model) does not sufficiently explain the high GD rates exhibited by these genomes, even after accounting for the singularities of the *M. leprae* lineage (GD-PRl-ML model)

**Table 1:** Results of the Branch Models of Gene Turnover Fitted to the Gene Families Present in the Genus *Mycobacterium*

Branch Model	No. of Parameters	Log-Likelihood Score	AIC Score	ΔAIC
GD-GR-ML	3	−90,718.03	181,442.06	29,783.16
GD-PR-ML	5	−89,185.81	178,381.62	26,722.72
GD-PRl-ML	7	−87,408.80	174,831.60	23,172.70
GD-FR-ML	69	−75,756.45	151,650.90	0.00

**Fig. 5.—:**
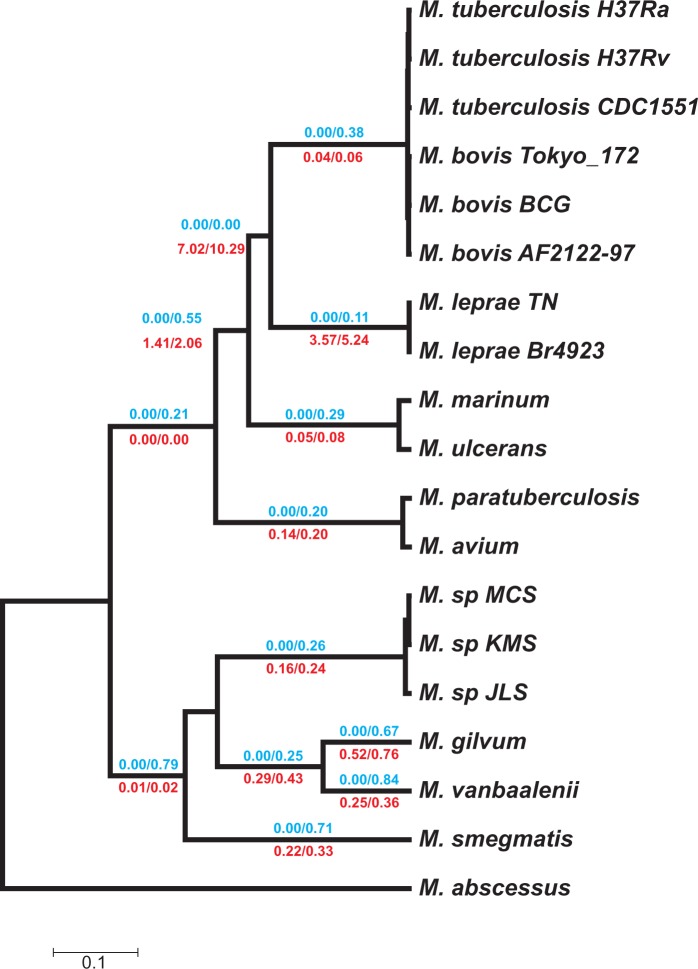
Gene turnover rates for each lineage under the GD-FR-ML+dG2 model. The numbers in branches indicate the two lineage-specific gain (in blue) and death (in red) rates estimated using the two discrete Gamma categories that account for the heterogeneity among gene families. The GD rates are expressed in events per amino acid substitution and events per gene and per amino acid substitution, respectively.

To understand the biological meaning of such high gene turnover rates, we analyzed the putative heterogeneity of GD rates across gene families. We calculated the likelihood of the data under a model that takes into account rate heterogeneity both across lineages and gene families (GD-FR-ML+dG2), which fitted the observed data significantly better than the FR model (ΔAIC = 2,158.13). Interestingly, the discrete Gamma distributions are leptokurtic (shape parameters of α_gain_ = 0.08, α_death_ = 0.99), indicating that GD processes are also heterogeneously distributed among families (fig. 5).

**Fig. 6.—:**
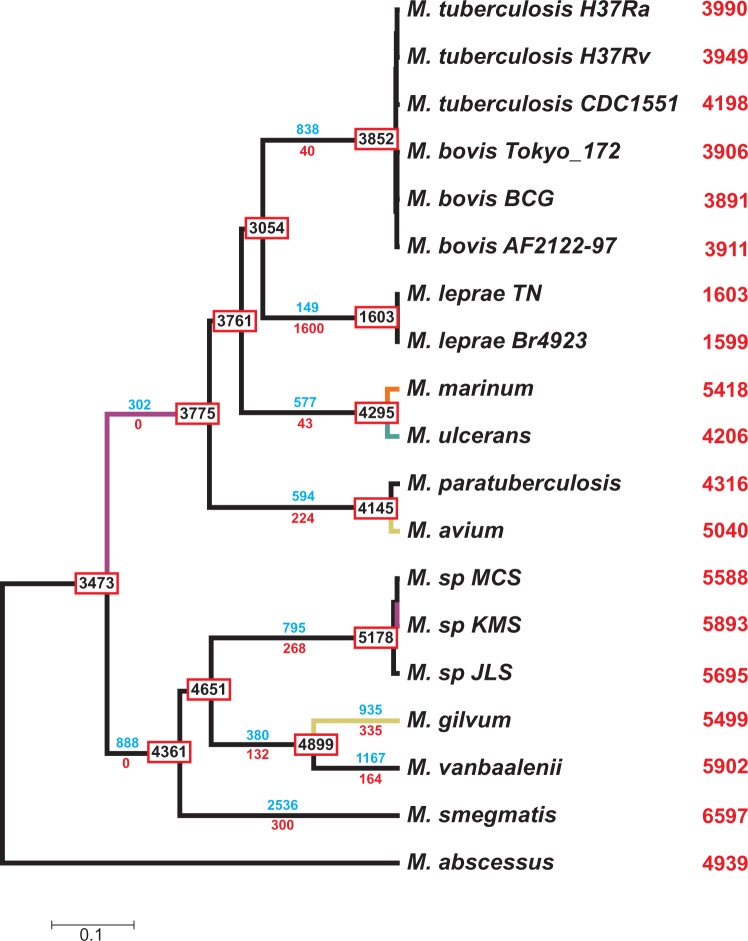
GD events under the best-fit model (GD-FR-ML+dG2). Numbers in internal nodes indicate the number of ancestral genes. Numbers on the branches denote the minimum number of gains (blue) and losses (red) estimated from the data. Red numbers on the right indicate the extant number of genes in each genome analyzed. Green, yellow, orange, and purple branches indicate the lineages where the outlier families (GD rates) are enriched in the biological processes of carbohydrate transmembrane transport, transposition, fatty acid biosynthesis, and the siderophore biosynthetic process, respectively.

Combining phylogenomic and gene gain-and-loss information, we estimated that the most recent common ancestor of the MTB clade likely had an intermediate number of genes (3,053) than previously predicted (2,977 [Gomez-Valero et al. 2007] and 3,901 [McGuire et al. 2012]). Our analysis demonstrates that despite sharing a very similar number of genes (especially for closely related species and subspecific strains), the species-specific gene repertoire is very different; for instance, the genomes of *M. ulcerans* and *M. tuberculosis*, harboring 4,206 and 3,990 genes, respectively, only share approximately half (48.30%) of their genes. Results also show that a global trend of gene number increases across the diversification of the *Mycobacterium* taxa (fig. 6). Indeed, when excluding both *M. leprae* and short (likely unreliable) branches, no lineage exhibits a clear net reduction in gene content. Remarkably, the nonpathogenic *Mycobacterium* lineages appear to have gained many more genes than the pathogenic ones. This effect is not explained by large expansions in specific branches, but is rather equally observed in both internal and external branches of this clade.

To gain insight into the functional meaning of such high gene turnover rates, we analyzed the overrepresented GO term categories among outlier families (i.e., particular families with rates that significantly depart from the estimated GD process in a given lineage). Given the best-fit model (GD-FR-ML+dG2), we found outlier gene families in 15 of the 34 lineages (supplementary table S2, Supplementary Material online), in both internal and external branches. Notably, most of the outlier gene families (33) displayed gain rates that were significantly higher than expected (on a given phylogenetic branch), whereas only one was significantly contracted. Specific GO categories (for biological processes) were found to be statistically overrepresented in 6 of the 34 analyzed branches (fig. 6), with transposition/DNA recombination and fatty acid biosynthesis being the most frequent GO term (found in two lineages each). This result points to transposable elements and fatty acid metabolism as the most important determinants of high gene turnover rates in mycobacteria.

Interestingly, some of the above-mentioned outlier gene families with overrepresented GO terms have been previously related to virulence in these bacteria (fig. 6) (Forrellad et al. 2013). In particular, gene families that encode important integral membrane components of the cell wall (NanT) (Ioerger et al. 2013), polyketide synthases (Pks) (Li et al. 2010), and enzymes responsible for the assembly of mycobactin (Mbt) (Quadri et al. 1998) displayed higher than expected gain rates in three of the pathogenic lineages. Among outlier families without significantly overrepresented GO terms (or with overrepresented terms of molecular function or cellular component), some *PE/PPE-* and *mmpL*-related genes (Domenech et al. 2005; Mukhopadhyay and Balaji 2011) exhibited higher gain rates in *M. marinum*. On the other hand, a family of genes related to fatty acid hydrolases (*Mmcs1433*), some endonucleases (genes related to *Mvan0273*), haloacetate dehalogenases (genes related to *Msmeg1984* and *Msmeg2340*), and hydrolases (genes related to *Mmar2844*) were outliers of gain rates in some lineages of the nonpathogenic clade.

## Discussion

Our comparative genomic analysis of mycobacteria improves upon the knowledge provided by previous studies that have focused on this group. First, we have estimated a specific amino acid replacement matrix (MYC) to obtain a more robust phylogeny, which is critical to study the gene content and family evolution in a phylogenetic context. Second, to study such mycobacteria genome evolution, we applied—for the first time—stochastic models of gene GD, which provide a sound probabilistic framework to contrast the fit of different evolutionary scenarios, and to separately estimate gene GD rates as well as ancestral gene content.

### Phylogenetic Relationships

Our phylogenomic framework not only allows for the proposal of a robust evolutionary scenario for the diversification of mycobacteria but also provides a trustworthy tree for subsequent phylogenetic analyses. In our ML tree, the different strains of *M. tuberculosis* and *M. bovis* form a single, well-defined clade, and pathogenic and nonpathogenic lineages are clearly separated. Nevertheless, both under a partitioned and an unpartitioned scheme, we uncovered some important topological differences with respect to most of the relationships published for this genus (Smith et al. 2012), specially the position of non-MTBC species (*M. marinum*, *M. ulcerans*, and *M. avium*) and of some nonpathogenic species. These topological differences seem to be data-driven as our 16S rRNA ML tree (supplementary fig. S2, Supplementary Material online) recovered a different topology. This locus has been considered as a “gold standard” for bacterial molecular systematics (Janda and Abbott 2007) since the days of DNA-DNA hybridization, but in spite of its functional and sequence conservation, it is now apparent that in the case of *Mycobacterium* as a genus, it does not reflect genome-level evolution. Four main topological differences emerged from the comparison of our ML tree and the 16S rRNA tree: 1) The separation of the *leprae–avium* clade (sister to MTBC) and the placement of the *avium* clade basal to all other slow growers, 2) the transfer of the *marinum–ulcerans* clade from a sister relationship with *M. leprae* to a more basal position outside the *M. leprae*–MTBC clade, 3) *M. smegmatis* was repositioned from sister taxon of the free-living soil strains to the base of the rapid growers, and 4) *M. gilvum* and *M. vanbaalenii* were pulled together in a well-supported clade sister to the soil strains. Our ML model-based approach also differs from Vishnoi et al. (2010) in the placement of *M. leprae* and the non-MTBC pathogenic species and from McGuire et al. (2012) in the phylogenetic relationships between *M. smegmatis* and the rest of nonpathogenic mycobacteria. The choice of amino acid substitution model did not impact topological inference, but our estimated MYC GTR model yielded a better likelihood score. Interestingly, our phylogenomic tree was not fully supported by the individual ortholog groups. As many other studies spanning a variety of organisms from fungi to vertebrates (Gatesy and Baker 2005; Salichos and Rokas 2013), we found that some orthologs exhibit a conflicting phylogenetic history when compared with each other, or even with the concatenated alignment-based phylogenomic tree. Although shallow nodes are supported by the vast majority of orthologs, deeper alternative groupings emerge when individual locus trees are contrasted. Concatenation produced a fully bootstrap-supported that masks the individual tree-to-tree disagreements. Reasons for such incongruity include stochasticity, for example, incomplete lineage sorting, varying sequence convergence levels, lack of phylogenetic informativeness, recombination, as well as HGT. By combining concatenation with locus-specific phylogenetic methods we were able to propose a robust total-evidence evolutionary scenario, while dissecting contradictory evolutionary signals at the gene level.

### Gene Content-Based Analysis

Although they are highly conserved at the sequence level throughout the genus, the number identifiable 1:1 orthologs in *Mycobacterium* taxa is very low. This pattern reflects the major impact of gene content changes, rather than amino acid substitutions, in genome evolution. This is especially true for the MTBC, where our gene-content phylogeny provides much higher resolution than the amino acid substitution matrix-based approach. Here, our analysis clearly supports (with high bootstrap support values) the origin of the *M. bovis* strains from a *M. tuberculosis*-like ancestor, being the H37Rv genome as the closest relative in terms of gene content. The topology for the rest of the genus is identical to the ML tree, confirming the inferences from our phylogenomic analysis and supporting our topology in contrast to the inferred in other studies (Vishnoi et al. 2010; McGuire et al. 2012).

### GD Dynamics

To study in more detail the gene dynamics across the genus, we applied a full likelihood method to study *Mycobacterium* genome evolution. The applied models not only allow for the decoupled estimation of gene GD rates (i.e., as two independently estimated parameters, γ and δ) but also explicitly take into account key features of the evolution of *Mycobacterium* species, such as HGT (i.e., gain rates that include new copies that originated from 0 ancestral genes; fig. 1) and, more importantly, the γ and δ heterogeneities across lineages and gene families. The use of these ML-based models allows the statistical evaluation of competing evolutionary scenarios and the selection of the one that best explains observed gene dynamics.

In this study, we demonstrate that *Mycobacterium* species show high gene turnover rates that differ markedly across lineages and families (GD-FR-ML+dG2 is the best fit model) and not merely between pathogenic and nonpathogenic lineages, like several previous studies assumed in their analyses (McGuire et al. 2012; Prasanna and Mehra 2013). Clearly, ignoring these rate heterogeneities can greatly bias estimates and the identification of particular outlier lineages and families, especially when using parsimony-based approaches. We found that our ML estimates of the number of genes at the ancestral nodes clearly differ with respect to that found by a previous study (McGuire et al. 2012), which were based on a gene trees–species tree reconciliation method implemented in the SYNERGY algorithm (Wapinski et al. 2007). These strong differences can likely be explained by the use of a more realistic (and complex) model under a solid statistical framework. We cannot rule out the possibility, however, that the previously reported low phylogenetic coverage of non-mycobacterial *Actinobacteria* (McGuire et al. 2012) could also have a significant influence on their results. In fact, the discordance among gene trees revealed by the network approach (or any other the potential bias leading to inaccurate branch lengths) may affect the downstream analysis, including the inference of GD rates and the ancestral gene content. This might especially affect estimates of the number of genes in the *M. leprae*–MTB ancestor, a node supported by a low number of individual gene trees.

The analysis of gene families with extreme GD rates may also be sensitive to the model assumed. In fact, the number of genes can differ between species simply by the stochastic turnover process, that is, without significant rate changes. Therefore, the mere comparison of family sizes is not sufficient to detect important family expansions or contractions. Our methodology, however, allows for the detection of these outliers within the estimated GD stochastic background. In fact, we have been able to identify specific lineages in which the gain or loss of gene family members might have significant biological relevance. Noticeably, we found manifest differences in the functional profile of outlier families between pathogenic and nonpathogenic lineages, with the latter presenting outlier families related to pathogenesis in *M. tuberculosis*. It has been documented that lipid metabolism is a major determinant of virulence in the MTBC either through its role in shaping the proteins and lipids that form the cell wall or in other cell surface modulation independent processes (Sonawane et al. 2012; Forrellad et al. 2013; Reddy et al. 2013). These virulence factors serve as targets for antimicrobial drug development (Tomioka et al. 2011; Wang et al. 2013; North et al. 2013). Here, we found that the most important gene gains in fatty acid and siderophore biosynthesis (Campbell and Cronan 2001; Rodriguez 2006) and carbohydrate membrane transport (Titgemeyer et al. 2007; Niederweis 2008) likely took place in the ancestral lineage that led to the pathogenic clade, after the split of *M. marinum* and *M. ulcerans*. Despite their close phylogenetic relationship, the two branches connecting *M. marinum* and *M. ulcerans* had accumulated gene families with unexpectedly high gene dynamism. Some of these gene families likely contributed to the important phenotypic differences that are observed between these two species (Yip et al. 2007; Stinear et al. 2008). The most dynamic gene families in nonpathogenic strains are enzymes (e.g., metal-dependent hydrolases, haloacetate dehalogenases, endonucleases, isopentenyl pyrophosphate isomerases) and transcriptional regulator encoding families. These results suggest that the diversification and adaptation of nonpathogenic mycobacteria to their different life-styles may have been promoted by changes in some of these gene families.

## Supplementary Material

Supplementary tables S1 and S2 and figures S1–S3 are available at *Genome Biology and Evolution* online (http://www.gbe.oxfordjournals.org/).

Supplementary Data
