# Supplementary material for: Mycobacterial Phylogenomics: An Enhanced Method for Gene Turnover Analysis Reveals Uneven Levels of Gene Gain and Loss among Species and Gene Families
Source: Genome Biol Evol. 2014 Jun 5;6(6):1454–65. doi: 10.1093/gbe/evu117 (PMC4079203; doi:10.1093/gbe/evu117)
Supplement: Supplementary Data [file supp_6_6_1454__index.html]

Mycobacterial phylogenomics: An enhanced method for gene turnover analysis reveals uneven levels of gene gain and loss among species and gene families — Mycobacterial Phylogenomics: An Enhanced Method for Gene Turnover Analysis Reveals Uneven Levels of Gene Gain and Loss among Species and Gene Families — Supplementary Data 

# Mycobacterial Phylogenomics: An Enhanced Method for Gene Turnover Analysis Reveals Uneven Levels of Gene Gain and Loss among Species and Gene Families

## Supplementary Data

files

**Files in this Data Supplement:**

- Supplementary Data - pdf file
- Supplementary Data - pdf file
- Supplementary Data - xls file
