# Supplementary material for: Mycobacterial Phylogenomics: An Enhanced Method for Gene Turnover Analysis Reveals Uneven Levels of Gene Gain and Loss among Species and Gene Families
Source: Genome Biol Evol. 2014 Jun 5;6(6):1454–65. doi: 10.1093/gbe/evu117 (PMC4079203; doi:10.1093/gbe/evu117)
Supplement: Supplementary Data [file supp_evu117_SupFigures.pdf]

Supplementary Figures

FIG. S1. Properties of the GTR MYC amino acid substitution matrix and comparison with other matrices. (A) Bubble plot of the exchangeability rate differences between the WAG (blue) and MYC (red) amino acid substitution matrices. (B) Comparison of amino acid frequencies among different matrices (MYC, WAG, LG, JTT).

FIG. S2. Topological differences between the phylogenetic reconstruction based on the concatenate of protein alignments and the commonly used bacterial marker locus 16S rRNA. Incongruences between trees are represented as red circles connected with red lines. Putative 16S rRNA duplications are indicated with red arrows (concatenated alignment-based tree) connected with red circles (16S rRNA tree). Values on the nodes indicate the bootstrap support values expressed as the proportion (%) of bootstrap trees that agree with a given bipartition on the best ML tree.

FIG. S3. Number of genes estimated in ancestral nodes under the four branch-specific gain and death rate models. GD-GR-ML, global rates model; GD-PR-ML, pathogenic lineage-specific rates model; GD-PRl-ML, GD-PR-ML model + *M. leprae* specific rates model; GD-FR-ML, free rates model.

Fig. S1

A

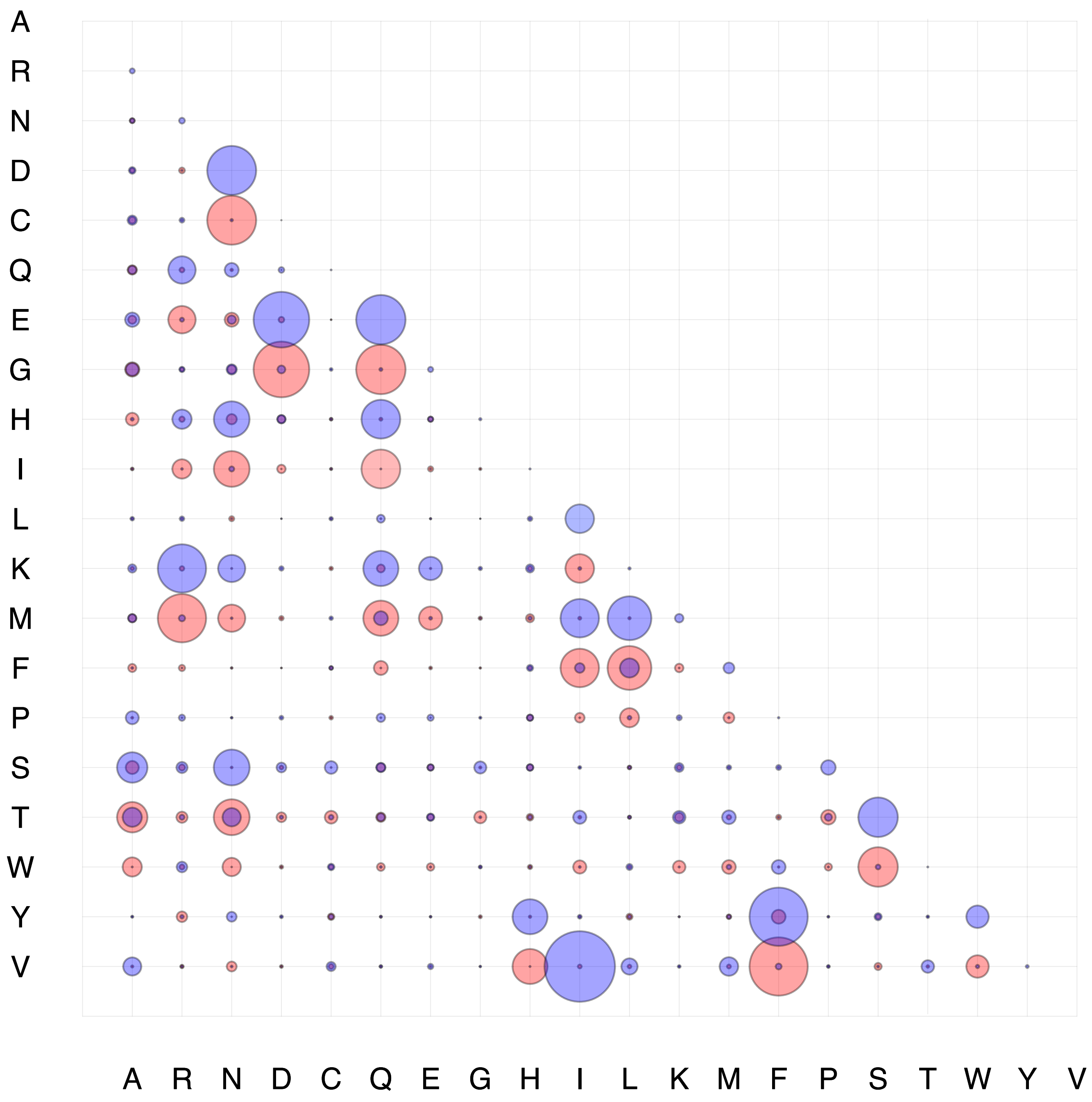
A
R
N
D
C
Q
E
G
H
I
L
K
M
F
P
S
T
W
Y
V
A R N D C Q E G H I L K M F P S T W Y V

B

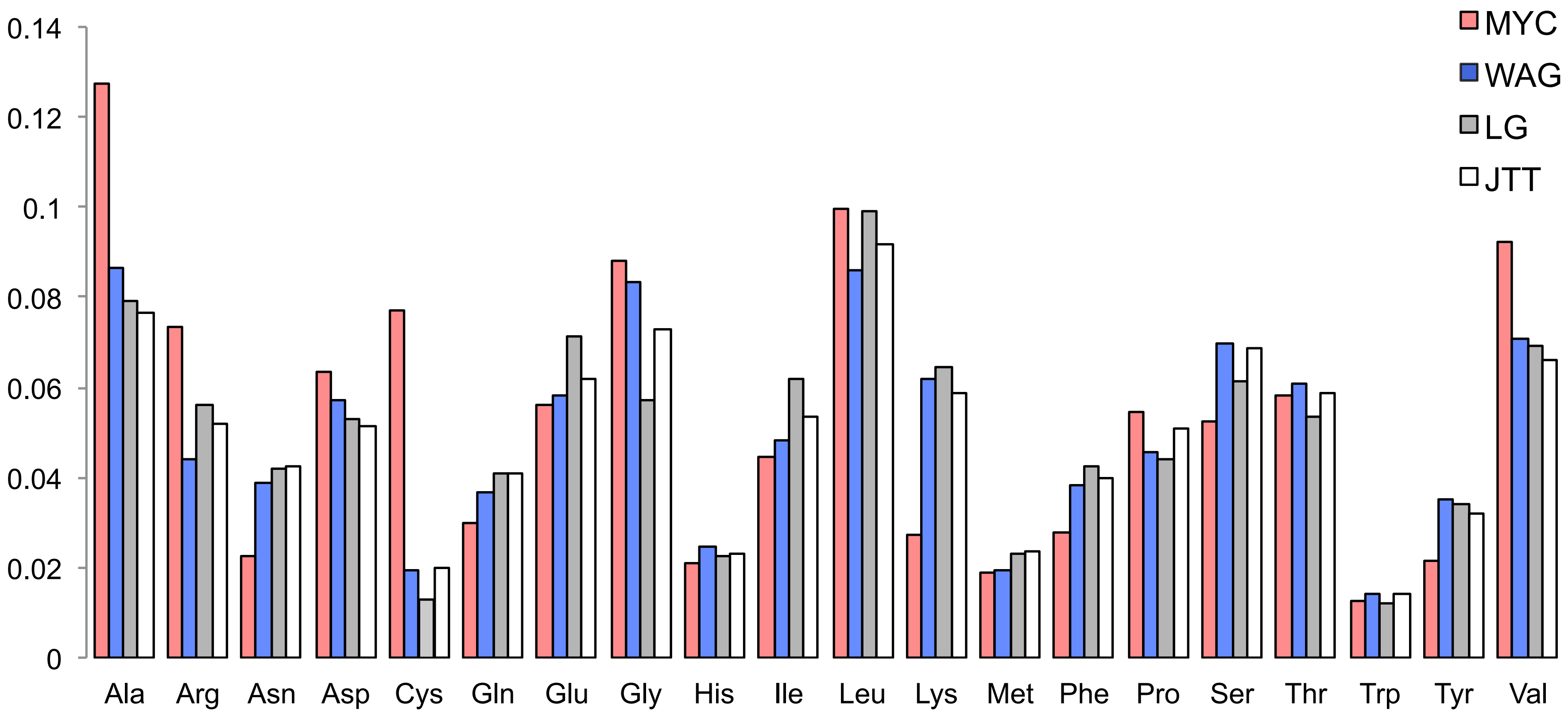
0.14
0.12
0.1
0.08
0.06
0.04
0.02
0
MYC
WAG
LG
JTT
Ala Arg Asn Asp Cys Gln Glu Gly His Ile Leu Lys Met Phe Pro Ser Thr Trp Tyr Val

Fig. S2

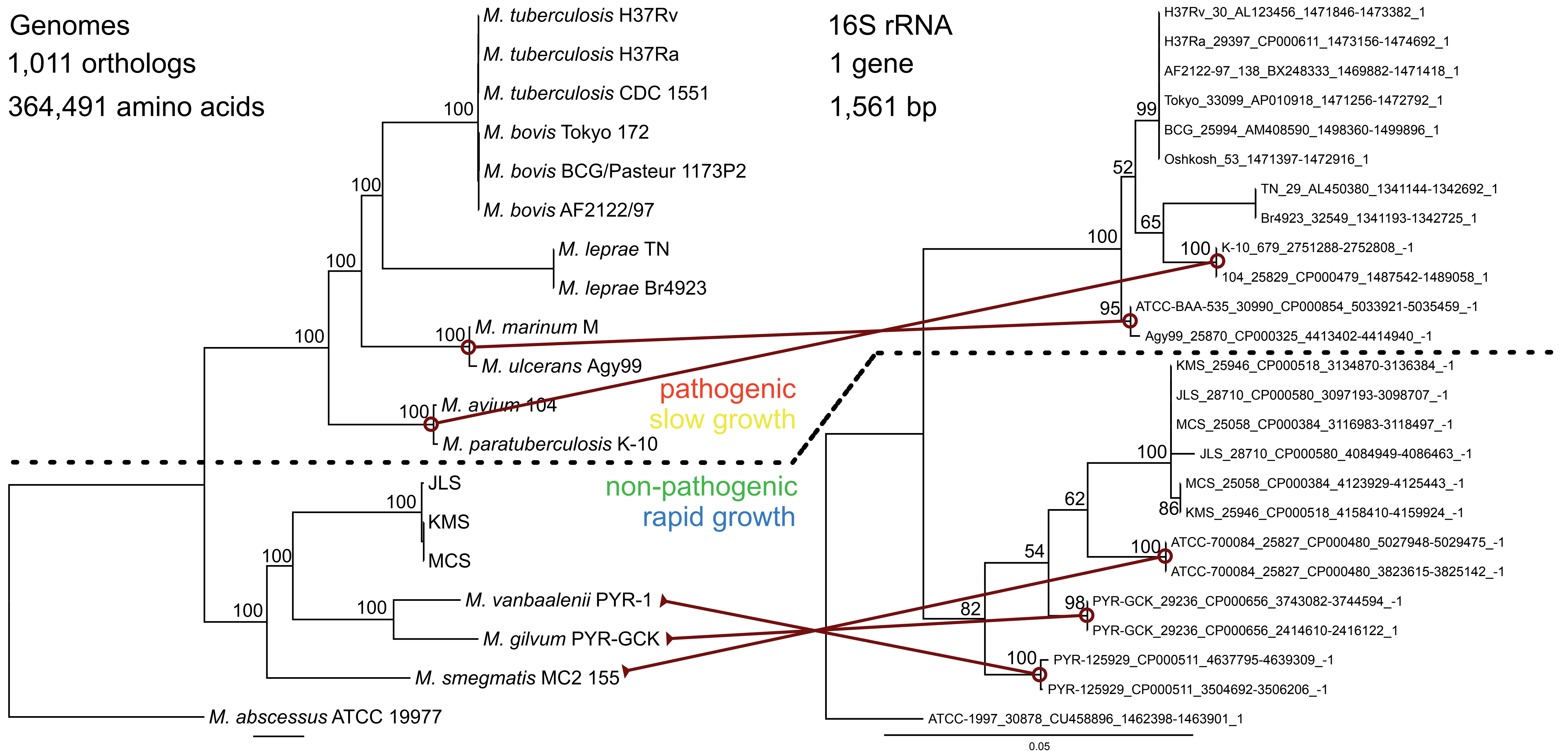
Genomes
1,011 orthologs
364,491 amino acids
M. tuberculosis H37Rv
M. tuberculosis H37Ra
M. tuberculosis CDC 1551
M. bovis Tokyo 172
M. bovis BCG/Pasteur 1173P2
M. bovis AF2122/97
M. leprae TN
M. leprae Br4923
M. marinum M
M. ulcerans Agy99
M. avium 104
M. paratuberculosis K-10
JLS
KMS
MCS
M. vanbaalenii PYR-1
M. gilvum PYR-GCK
M. smegmatis MC2 155
M. abscessus ATCC 19977
pathogenic
slow growth
non-pathogenic
rapid growth
16S rRNA
1 gene
1,561 bp
H37Rv_30_AL123456_1471846-1473382_1
H37Ra_29397_CP000611_1473156-1474692_1
AF2122-97_138_BX248333_1469882-1471418_1
Tokyo_33099_AP010918_1471256-1472792_1
BCG_25994_AM408590_1498360-1499896_1
Oshkosh_53_1471397-1472916_1
TN_29_AL450380_1341144-1342692_1
Br4923_32549_1341193-1342725_1
K-10_679_2751288-2752808_-1
104_25829_CP000479_1487542-1489058_1
ATCC-BAA-535_30990_CP000854_5033921-5035459_-1
Agy99_25870_CP000325_4413402-4414940_-1
KMS_25946_CP000518_3134870-3136384_-1
JLS_28710_CP000580_3097193-3098707_-1
MCS_25058_CP000384_3116983-3118497_-1
JLS_28710_CP000580_4084949-4086463_-1
MCS_25058_CP000384_4123929-4125443_-1
KMS_25946_CP000518_4158410-4159924_-1
ATCC-700084_25827_CP000480_5027948-5029475_-1
ATCC-700084_25827_CP000480_3823615-3825142_-1
PYR-GCK_29236_CP000656_3743082-3744594_-1
PYR-GCK_29236_CP000656_2414610-2416122_1
PYR-125929_CP000511_4637795-4639309_-1
PYR-125929_CP000511_3504692-3506206_-1
ATCC-1997_30878_CU458896_1462398-1463901_1
0.05

Fig. S3

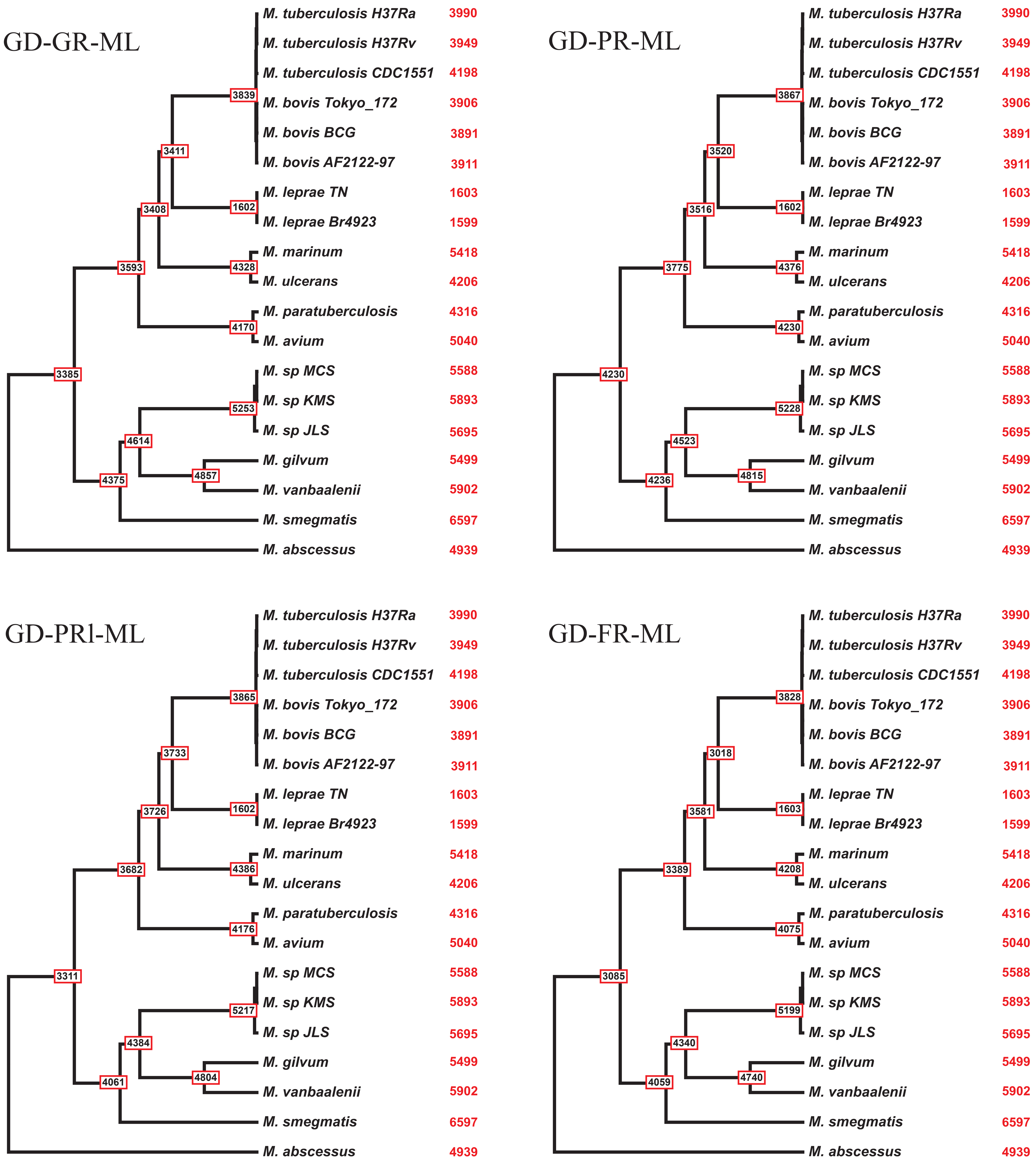
GD-GR-ML
M. tuberculosis H37Ra 3990
M. tuberculosis H37Rv 3949
M. tuberculosis CDC1551 4198
M. bovis Tokyo_172 3906
M. bovis BCG 3891
M. bovis AF2122-97 3911
M. leprae TN 1603
M. leprae Br4923 1599
M. marinum 5418
M. ulcerans 4206
M. paratuberculosis 4316
M. avium 5040
M. sp MCS 5588
M. sp KMS 5893
M. sp JLS 5695
M. gilvum 5499
M. vanbaalenii 5902
M. smegmatis 6597
M. abscessus 4939
3839
3411
3408
1602
3593
4328
4170
3385
5253
4614
4857
4375
GD-PR-ML
M. tuberculosis H37Ra 3990
M. tuberculosis H37Rv 3949
M. tuberculosis CDC1551 4198
M. bovis Tokyo_172 3906
M. bovis BCG 3891
M. bovis AF2122-97 3911
M. leprae TN 1603
M. leprae Br4923 1599
M. marinum 5418
M. ulcerans 4206
M. paratuberculosis 4316
M. avium 5040
M. sp MCS 5588
M. sp KMS 5893
M. sp JLS 5695
M. gilvum 5499
M. vanbaalenii 5902
M. smegmatis 6597
M. abscessus 4939
3867
3520
3516
1602
3775
4376
4230
4230
5228
4523
4815
4236
GD-PRl-ML
M. tuberculosis H37Ra 3990
M. tuberculosis H37Rv 3949
M. tuberculosis CDC1551 4198
M. bovis Tokyo_172 3906
M. bovis BCG 3891
M. bovis AF2122-97 3911
M. leprae TN 1603
M. leprae Br4923 1599
M. marinum 5418
M. ulcerans 4206
M. paratuberculosis 4316
M. avium 5040
M. sp MCS 5588
M. sp KMS 5893
M. sp JLS 5695
M. gilvum 5499
M. vanbaalenii 5902
M. smegmatis 6597
M. abscessus 4939
3865
3733
3726
1602
3682
4386
4176
3311
5217
4384
4804
4061
GD-FR-ML
M. tuberculosis H37Ra 3990
M. tuberculosis H37Rv 3949
M. tuberculosis CDC1551 4198
M. bovis Tokyo_172 3906
M. bovis BCG 3891
M. bovis AF2122-97 3911
M. leprae TN 1603
M. leprae Br4923 1599
M. marinum 5418
M. ulcerans 4206
M. paratuberculosis 4316
M. avium 5040
M. sp MCS 5588
M. sp KMS 5893
M. sp JLS 5695
M. gilvum 5499
M. vanbaalenii 5902
M. smegmatis 6597
M. abscessus 4939
3828
3018
3581
1603
3389
4208
4075
3085
5199
4340
4740
4059
