# Supplementary material for: Mycobacterial Phylogenomics: An Enhanced Method for Gene Turnover Analysis Reveals Uneven Levels of Gene Gain and Loss among Species and Gene Families
Source: Genome Biol Evol. 2014 Jun 5;6(6):1454–65. doi: 10.1093/gbe/evu117 (PMC4079203; doi:10.1093/gbe/evu117)
Supplement: Supplementary Data [file supp_evu117_TableS1.pdf]

Table S1. Data sets used for the analyses

Data set	Analysis	Genomes	Markers and data information
**Myc19**	Phylogenetic reconstruction	The 19 mycobacteria proteomes (*n* = 19)<br><br>Data Set	Concatenation of 1,011 aligned protein sequences (1:1 orthologs; 364,491 amino acid positions)<br>Data Set<br>MYC amino acid substitution matrix
**Myc18**	Gain and death analysis	The Myc19 data set, excluding *M. abscessus* (*n* = 18)	14,108 ortholog groups (or gene families; N:N and N:M relationships)
